# A Power Amplifier with Large High-Efficiency Range for 5G Communication

**DOI:** 10.3390/s20195581

**Published:** 2020-09-29

**Authors:** Zhiwei Zhang, Zhiqun Cheng, Guohua Liu

**Affiliations:** School of Electronics and Information, Hangzhou Dianzi University, Hangzhou 310018, China; zzw@hdu.edu.cn (Z.Z.); ghliu@hdu.edu.cn (G.L.)

**Keywords:** Doherty power amplifier, drain-to-source capacitance, large efficiency range, 5G communication

## Abstract

This paper presents a new method to design a Doherty power amplifier (DPA) with a large, high-efficiency range for 5G communication. This is through analyzing the drain-to-source capacitance (*C*_DS_) of DPAs, and adopting appropriate impedance of the peak device. A closed design process is proposed, to design the extended efficiency range DPA based on derived theories. For validation, a DPA with large efficiency range was designed and fabricated by using two equal devices. The measured results showed that the saturated output power was between 43.4 dBm and 43.7 dBm in the target band. Around 70% saturated drain efficiency is obtained with a gain of greater than 11 dB. Moreover, the obtained drain efficiency is larger than 50% at the 10 dB power back-off, when operating at 3.5 GHz. These superior performances illustrate that the implemented DPA can be applied well in 5G communication.

## 1. Introduction

With the rapid development of communication technology, the amount of data transferred worldwide is increasing significantly [1]. Limited spectrum resources have become extremely precious. There are many methods to improve the efficiency of spectrum transmission. Modern wireless communication signals usually adopt complex modulation methods to improve the utilization efficiency of spectrum resources [2,3]. These signals are often large peak-to-average ratio signals, and traditional power amplifiers (PAs) are not suitable for amplifying these signals [4,5]. Therefore, higher performance is required from the power amplifiers.

Some power amplifiers for high peak-to-average ratio signals are proposed, such as Doherty [6], out-phasing [7], and envelope tracking [8]. Among them, Doherty PAs (DPAs) are widely used in practical base stations because of their simple structure and low cost. However, DPAs also have some inherent disadvantages, such as a narrow bandwidth and only 6 dB output power back-off (OBO) in traditional symmetrical DPAs [9,10]. It is apparent that a 6 dB OBO cannot meet current communication requirements. In order to extend the OBO range, many methods have been proposed [11,12,13,14,15,16]. In [11,12], a multiway is applied to extend OBO, which uses multiple peaking PA branches that turn on at various power levels to provide multiple efficiency peaks at and beyond 6 dB OBO. Asymmetrical topology is adapted to ensure the peak PA has a larger saturated current than that of the carrier PA, to increase OBO [13,14]. However, the multiway and asymmetrical architectures often increase design complexity and under-utilize the power capacity of active devices.

Recently, dual-input DPAs have been proposed to extend the OBO range by realizing wider load modulation, which relies on dynamically adjusting the phase and amplitude of the input signal by using digital techniques [15,16]. However, dual-input DPAs with digital controlling techniques suffer from increased circuit complexity, and/or higher manufacturing costs.

Many efficiency range extension methods are also presented in symmetrical architecture [17,18,19,20,21,22]. In [17], a symmetrical DPA with complex impedance at the DPA combiner is demonstrated to achieve larger load modulation compared to a conventional symmetrical DPA. High efficiency over large dynamic ranges is achieved using symmetrical devices, while still maintaining full voltage and current utilization of both transistor cells. This is achieved by modifying the combiner [18]. In [19], an explicit circuit model of generalized symmetrical DPA is proposed to design DPAs with an extended high-efficiency range. A methodology is proposed for extending the high-efficiency power range of symmetrical DPAs by taking advantage of the output impedance of peaking stage [20]. Recently, a symmetrical DPA with an extended efficiency range has been presented, where the phase relationship between the carrier and peaking currents at the DPA’s combiner is used to extend the dynamic load modulation range [21]. In [22], the transistor’s nonlinear phase distortion architecture is used to enhance the average drain efficiency of the DPA with the proper choice of carrier and peaking PA load trajectories. In the fore-mentioned papers, current, impedance and even phase distortion are analyzed and used to enhance efficiency of DPAs by improving the design. In practical design, some methods are introduced to eliminate the influence of drain-to-source capacitance, *C*_DS_. As described in [23,24], the quasi-lumped transmission line is used to absorb *C*_DS_ when designing the matching output network. In [25], the quarter-wave impedance inverter is approximated by the internal and packaged elements of transistors, together with carrier matching network. However, DPAs’ theories with *C*_DS_ are not analyzed and derived in detail. Unlike the above papers, in this work, we are not committed to eliminating the impact of *C*_DS_ on DPA performance, although we have developed a method to use *C*_DS_ to achieve a larger OBO range. In other words, the existence of *C*_DS_ can be positive for expanding the OBO range through proper design. In [26], the nonlinear output capacitor of the transistor has been used to generate harmonic components, thus, improving the saturated drain efficiency. Different from [26], this work is aimed at a large range of high efficiency, including two levels of saturation and power back-off, not just the saturation level.

This paper briefly analyzes DPAs with the drain-to-source (*C*_DS_). Adopting suitable impedance of peak device is presented to extend the high-efficiency range, which depends on the existence of the *C*_DS_. The design parameters are derived in detail for DPAs with *C*_DS_. A closed design process is proposed to design extended efficiency range DPA based on the modified theories and two equal transistors. An extended efficiency range DPA is fabricated using two identical devices. The structure of this paper is as follows: the theories of DPAs with *C*_DS_ are analyzed in Section 2; the closed design process of the extended efficiency range DPA is described in detail in Section 3; simulated and measured results are analyzed in detail in Section 4; Section 5 summarizes the content of this paper.

## 2. Theory Analysis of The Proposed DPA

**Figure 1:**
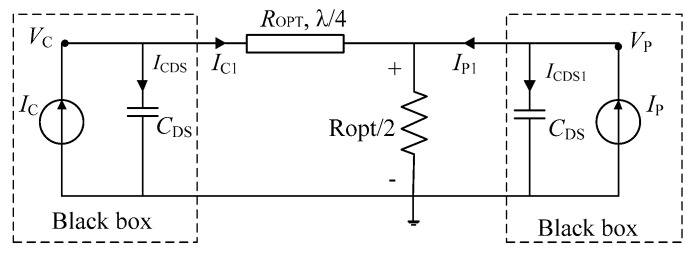
Conventional Doherty power amplifier (DPA) topology with drain-to-source capacitance (*C*_DS_).

The traditional DPA includes a carrier PA branch and a peak PA branch. At the combiner, there is a load of *R_OPT_*/2. A transistor can be equivalent to an ideal current source. The equivalent traditional DPA topology is shown in Figure 1. There is an impedance converter line in the carrier PA branch, as shown in Figure 1. In the analysis of conventional ideal DPAs, the drain-source capacitance *C*_DS_ is often ignored. In the practical design process, the drain-source capacitance *C*_DS_ is usually adopted into the output matching circuits to reduce its impact on the performance of the PA.

As shown in Figure 1, we consider the current source and drain-source capacitance *C*_DS_ as comprising a black box. Therefore, considering the voltage *V*_C_ and current *I*_C1_ outside the black box, the analysis method of traditional DPAs is still applicable. The only difference is that conventional DPAs have an ideal current source, and the current source in this paper is with a *C*_DS_.

**Figure 2:**
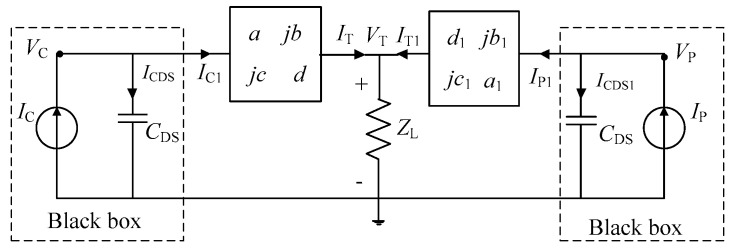
The proposed DPA topology.

Based on Figure 1, a more general schematic diagram is shown in Figure 2. The output matching networks (OMN) and offset lines of carrier and peak PAs are represented by two ABCD transfer matrices. We will derive the design theories of DPAs with *C*_DS_ using the topology shown in Figure 2. While deriving the theories of DPAs, the *C*_DS_ is considered as shown in Figure 2.

Based on Figure 2, the following equations can be obtained based on two-port network theories.

(1) $$\left[\begin{array}{c} V_{C}\\ I_{C} \end{array}\right]=\left[\begin{array}{cc} 1 & 0\\ Z_{CDS}^{-1} & 1 \end{array}\right]\left[\begin{array}{cc} a & jb\\ jc & d \end{array}\right]\left[\begin{array}{c} V_{T}\\ I_{T} \end{array}\right],$$

(2) $$\left[\begin{array}{c} V_{P}\\ I_{P} \end{array}\right]=\left[\begin{array}{cc} 1 & 0\\ Z_{CDS}^{-1} & 1 \end{array}\right]\left[\begin{array}{cc} d_{1} & jb_{1}\\ jc_{1} & a_{1} \end{array}\right]\left[\begin{array}{c} V_{T}\\ I_{T1} \end{array}\right]$$

Then, the voltage of the carrier device, *V_C_*, and the voltage of the peak device, *V_P_*, can be derived as

(3) $$V_{C}=\frac{AQ_{4}-A_{1}Q_{2}}{Q_{1}Q_{4}-Q_{2}Q_{3}}I_{C}+\frac{BQ_{4}-B_{1}Q_{2}}{Q_{1}Q_{4}-Q_{2}Q_{3}}I_{P}$$

(4) $$V_{P}=\frac{AQ_{3}-A_{1}Q_{1}}{Q_{2}Q_{3}-Q_{1}Q_{4}}I_{C}+\frac{BQ_{3}-B_{1}Q_{1}}{Q_{2}Q_{3}-Q_{1}Q_{4}}I_{P}$$

(5) $$\left[\begin{array}{cc} A & B\\ A_{1} & B_{1} \end{array}\right]=\left[\begin{array}{cc} \frac{aZ_{L}+jb}{jcZ_{L}+d} & \frac{Z_{L}/d_{1}}{jcZ_{L}+d}\\ \frac{Z_{L}}{d_{1}\left(jcZ_{L}+d\right)} & \frac{jb_{1}}{d_{1}}+\frac{a_{1}dZ_{L}}{d_{1}\left(jcZ_{L}+d\right)} \end{array}\right]$$

(6) $$\left[\begin{array}{cc} Q_{1} & Q_{2}\\ Q_{3} & Q_{4} \end{array}\right]=\left[\begin{array}{cc} 1+\frac{A}{Z_{CDS}} & \frac{B}{Z_{CDS}}\\ \frac{A_{1}}{Z_{CDS}} & 1+\frac{B_{1}}{Z_{CDS}} \end{array}\right]$$

The carrier branch current *I_T_* and the peak branch current *I*_*T*1_ at the combiner can be expressed as

(7) $$I_{T}=\left[W-WZ_{CDS}^{-1}\frac{AQ_{4}-A_{1}Q_{2}}{Q_{1}Q_{4}-Q_{2}Q_{3}}+YZ_{CDS}^{-1}\frac{AQ_{3}-A_{1}Q_{1}}{Q_{2}Q_{3}-Q_{1}Q_{4}}\right]I_{C}+\;\left[-Y+YZ_{CDS}^{-1}\frac{BQ_{3}-B_{1}Q_{1}}{Q_{2}Q_{3}-Q_{1}Q_{4}}\right]I_{P}\;$$

(8) $$I_{T1}=\left[\frac{A_{1}Q_{1}-AQ_{3}}{Z_{CDS}d_{1}\left(Q_{2}Q_{3}-Q_{1}Q_{4}\right)}\right]I_{C}-\left[\frac{1}{d_{1}}-\frac{1}{Z_{CDS}d_{1}}\frac{BQ_{3}-B_{1}Q_{1}}{Q_{2}Q_{3}-Q_{1}Q_{4}}\right]I_{P},$$

where $W=\frac{d_{1}}{d_{1}\left(jcZ_{L}+d\right)}$, $Y=\frac{jcZ_{L}}{d_{1}\left(jcZ_{L}+d\right)}$.

The load impedance of the carrier transistor *Z_C_* can be calculated as

(9) $$Z_{C}=\frac{V_{C}}{I_{C}}$$

A coefficient of *β* can be defined as

(10) $$\beta =\frac{Z_{C,BACK}}{Z_{C,SAT}},$$

where *Z_C,SAT_* and *Z_C,BACK_* represent the load impedance of the carrier transistor at the saturation and the OBO level, respectively.

**Figure 3:**
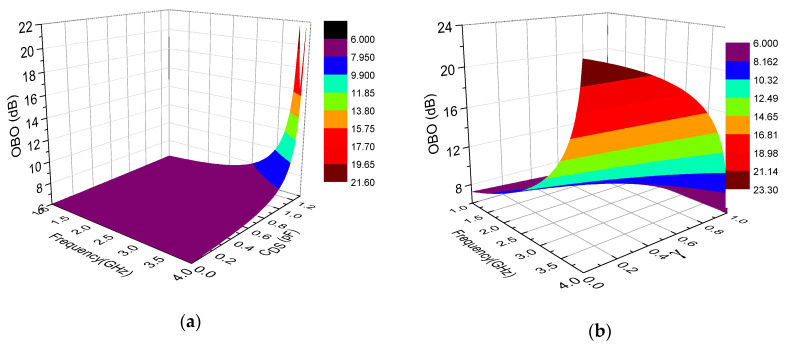

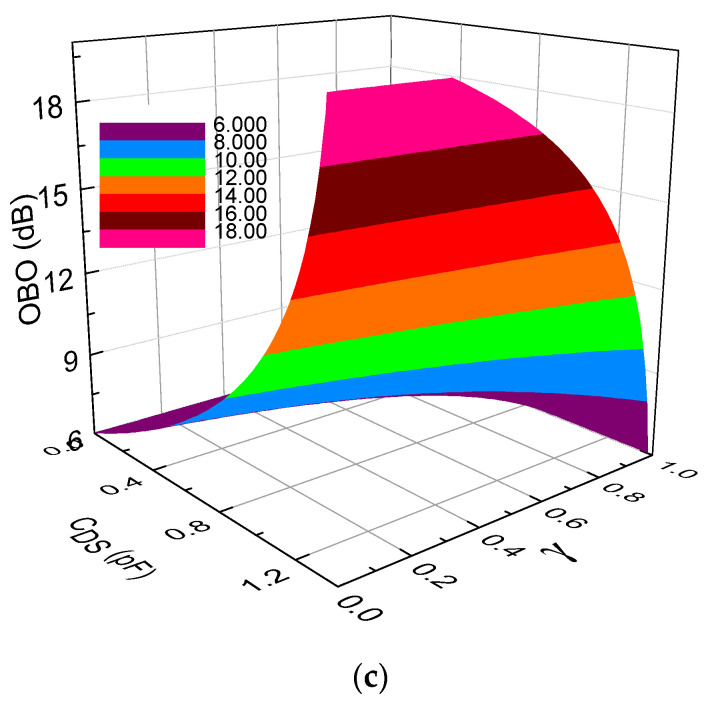
Relationships between output power back-off (OBO), coefficient *γ*, frequency and capacitance: (**a**) *γ* of 0.85, (**b**) capacitance of 1.22 pF, and (**c**) frequency of 3.5 GHz.

The OBO with *C*_DS_ effect included can be calculated as

(11) $$\mathrm{OBO}=10\log\left[\frac{P_{OUT,SAT}}{P_{OUT,BACK}}\right]=10\log\left[\left(2\beta \right)\left(\frac{I_{P,SAT}}{I_{C,SAT}}\right)\frac{\left(Z_{CDS}-V_{P,SAT}/I_{P,SAT}\right)}{\left(Z_{CDS}-V_{C,SAT}/I_{C,SAT}\right)}\right],$$

where *I_C,SAT_* and *I_P,SAT_* represent the saturated current of the carrier and the peak PA, respectively. From Equation (11), the $\frac{\left(Z_{CDS}-V_{P,SAT}/I_{P,SAT}\right)}{\left(Z_{CDS}-V_{C,SAT}/I_{C,SAT}\right)}$ is introduced due to the existence of *C*_DS_, which makes the OBO range more flexible in design parameters compared to OBO expression of traditional DPAs. In fact, OBO is also related to output capacitance. Furthermore, we can use different *Z*_CDS_ to obtain a different OBO. Figure 3 shows the relationships between OBO, coefficient *γ*, frequency and capacitance. To keep the OBO larger than 6 dB, the capacitance is up to 1.4 pF. In this paper, the DPA with a large OBO range is designed at 3.5 GHz. Therefore, the appropriate capacitance value and coefficient *γ* can be obtained from Figure 3.

For an ideal symmetrical DPA, the saturated current of carrier transistor *I_C,SAT_* should be equal to the saturated current of peak transistor *I_P,SAT_*. Therefore, Equation (11) can be simplified as

(12) $$\mathrm{OBO}=10\log\left[\left(2\beta \right)\frac{\left(Z_{CDS}-V_{P,SAT}/I_{P,SAT}\right)}{\left(Z_{CDS}-V_{C,SAT}/I_{C,SAT}\right)}\right]$$

In the design of conventional DPA, the $V_{P,SAT}/I_{P,SAT}$ is equal to $V_{C,SAT}/I_{C,SAT}$ that is *R_OPT_*. So, the OBO range expressed in Equation (12) is the same as that of traditional DPAs.

Here, the mentioned relationships should be defined as

(13) $$V_{C,SAT}/I_{C,SAT}=R_{OPT}$$

(14) $$V_{P,SAT}/I_{P,SAT}=\gamma R_{OPT},$$

where γ is a coefficient. Therefore, Equation (12) can be modified as

(15) $$\mathrm{OBO}=10\log\left[\left(2\beta \right)\frac{\left(Z_{CDS}-\gamma R_{OPT}\right)}{\left(Z_{CDS}-R_{OPT}\right)}\right]=10\log\left[\left(2\beta \right)\left(1+\frac{\left(R_{OPT}-\gamma R_{OPT}\right)}{\left(Z_{CDS}-R_{OPT}\right)}\right)\right]$$

From Equation (15), it is clear that the OBO would be larger than that of conventional DPAs when *γ* is between 0 and 1. A simple way to realize the *γR_OPT_* is to use different size transistors for the carrier and peak PA. However, choosing different transistors means asymmetric topology, which has some drawbacks as described in the introduction section. So, in this paper, the same devices in the carrier and the peak PA are used.

**Figure 4:**
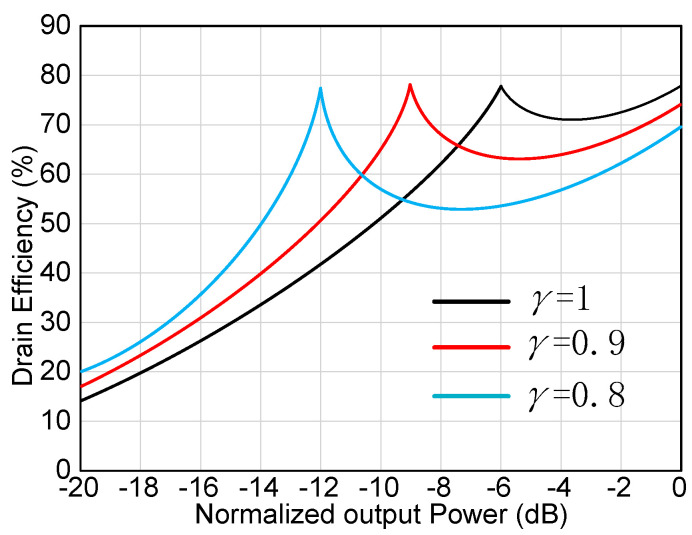
Drain efficiency versus normalized output power.

**Figure 5:**
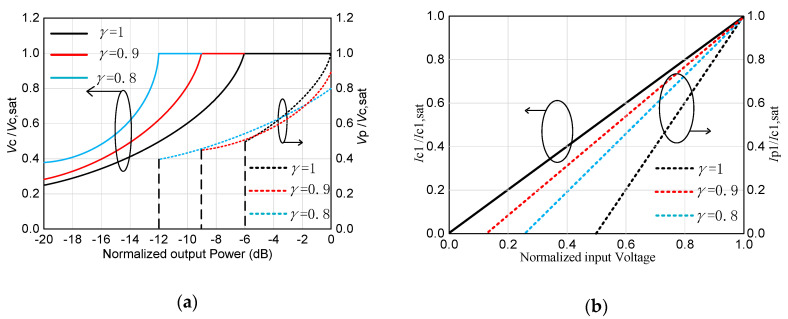
Voltage and current of the carrier power amplifier (PA) and peak PA: (**a**) Voltage, and (**b**) Current.

For an ideal symmetrical DPA, *β* value being 2, the OBO is calculated and plotted in Figure 4 and Figure 5. Figure 4 shows the voltage and current of the carrier PA and peak PA. From Figure 4 and Figure 5, the OBO value is 6 dB when γ value is 1, which is the same as that of conventional DPAs. As *γ* decreases, the OBO range begins to extend. When *γ* decreases to 0.8, the OBO can ideally reach 12 dB. This back-off range is excellent. However, it is worth noting that the saturated output power of the peak PA will decrease when load impedance deviates from the optimal impedance *R_OPT_*. It can also be seen from Figure 5 that the drain efficiency at the saturated level is smaller than 78.5%, and the drain efficiency at the OBO level can reach 78.5%.

The output power $P_{OUT,P}$ of peak PA can be calculated as

(16) $$P_{OUT,P}=0.5\times {\left(\frac{I_{max}}{2}\right)}^{2}\times \gamma R_{OPT}$$

It can be seen that the output power is a linear function of *γ*. Because *γ* is less than 1, the output power of the peak PA would decline. For example, *γ* of 0.91 should be taken for realizing the OBO of 9 dB. At the same time, the output power of peak PA will be 10% less. Different applications would mean a different tendency. So, in practical design, compromise should be made between saturated output power and OBO.

In traditional DPAs, if matching to a different *γR_OPT_* resistance, the current of the peak PA and the carrier is usually different. Thus, the impedance modulation of combined nodes of DPA will be changed. Thereby, it becomes an asymmetrical DPA. In this paper the same transistor is used, however, the optimal load impedance of the peak PA is set to *γR_OPT_*. In order to ensure that the saturated current of the peak PA and the carrier PA is consistent, the voltage of the peak PA should be set to *γV*_max_. As seen from Figure 5, the current and voltage of the peak PA satisfy the desired value. It means that the saturated current of the peak PA is equal to that of the carrier PA. At the same time, the saturated voltage of the peak PA is equal to *γV*_max_. Thus, the impedance modulation of combine node of DPA is the same as that of the symmetrical DPA. Therefore, this proposed DPA is still called a symmetrical DPA despite different output power of carrier PA and peak PA.

**Figure 6:**
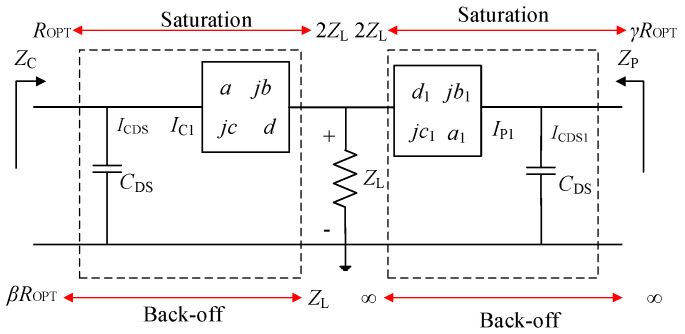
Impedance conversion of the DPA.

Figure 6 displays the impedance conversion of the DPA at the saturation and OBO level. In this paper, *I*_T_ is equal to *I*_T1_ and they are in phase. In saturation, impedance at the combiner seen from the carrier branch is 2*Z_L_*, and the load impedance *Z*_C_ of the carrier transistor is *R_OPT_*. The 2*Z_L_* is transformed to *Z*_C_ by using the (OMN)_C_ and the *C*_DS_. At the OBO level, the impedance at the combiner seen from the carrier branch is *Z_L_*, and the load impedance *Z_C_* of the carrier transistor is *βR_OPT_*. The *Z_L_* is also transformed to *βR_OPT_* by using the (OMN)_C_ and the *C*_DS_. The impedance at the combiner seen from the peak branch is 2*Z_L_*, and the load impedance *Z*_P_ of the peak transistor is *γR_OPT_* in saturation. The 2*Z_L_* is transformed to *R_OPT_* by using the (OMN)_P_ and the *C*_DS_. At the OBO level, the peak transistor is off, where the impedance *Z_P_* is infinite. The impedance at the combiner seen from the peak branch is also ∞. This impedance conversion is also achieved by using the (OMN)_P_ and the *C*_DS_.

Combining the impedance conversion with Equations (1)–(10), the (OMN)_C_ and (OMN)_P_ parameters can be derived as

(17) $$a=-\frac{R_{OPT}Z_{CDS}}{R_{OPT}+Z_{CDS}}\left(\beta -2\right)c,$$

(18) $$b=\frac{R_{OPT}Z_{CDS}}{R_{OPT}+Z_{CDS}}Z_{L}\left(2\beta -2\right)c,$$

(19) $$c=\sqrt{\left(2\beta -1\right)/\left[R_{OPT}Z_{L}\left(2\beta -2\right)\left(\beta +1\right)\right]},$$

(20) $$d=Z_{L}c\left(2\beta -2\right)/\left(2\beta -1\right),$$

(21) $$a_{1}=\sqrt{\frac{\gamma R_{OPT}Z_{CDS}}{\left(0.9R_{OPT}+Z_{CDS}\right)*2Z_{L}}},$$

(22) $$b_{1}=-\sqrt{2Z_{L}\left(\frac{\gamma R_{OPT}Z_{CDS}}{\gamma R_{OPT}+Z_{CDS}}\right)},$$

(23) $$c_{1}=0,$$

(24) $$d_{1}=\sqrt{2Z_{L}/\left(\frac{\gamma R_{OPT}Z_{CDS}}{\gamma R_{OPT}+Z_{CDS}}\right)}.\;$$

## 3. Design of the Proposed DPA

**Figure 7:**
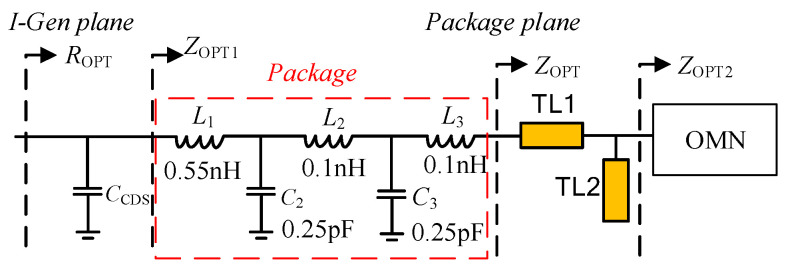
PA branch topology with device parameters.

In this section, a closed method is presented in detail to design the extended efficiency range symmetrical DPA based on the above-mentioned theories. In order to validate the proposed method, a DPA with extended efficiency range was designed using CGH40010F GaN HEMT, based on Rogers R4350B substrate. The drain voltage *V*_ds_ was set at 28 V. The gate voltage of the carrier PA *V*_gs1_ was −2.8 V and the gate voltage of the peak PA *V*_gs2_ was −5.7 V. The optimum load impedance *R*_OPT_ is determined as 32 Ω for CGH40010F considering *V*_knee_. Load-pull simulation should be processed in the ADS software to obtain the optimized load impedance *Z_OPT_* at the package plane, for deriving package parameters. Fortunately, the package parameters of CGH40010F can be found from [27], shown in Figure 7. As shown in Figure 7, the package parameters are included for consideration in the practical design process.

### 3.1. Output Matching Network Design

**Figure 8:**
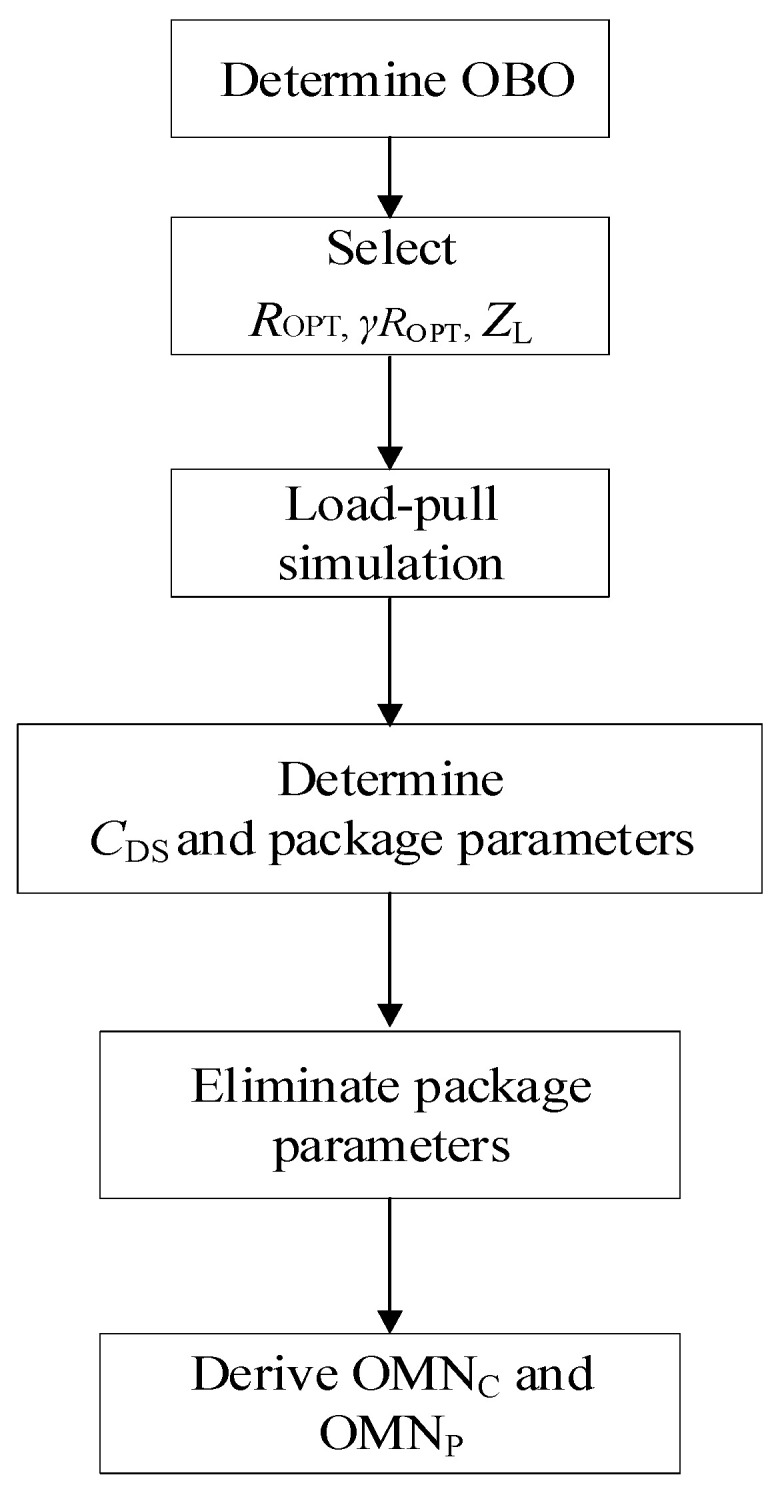
PA branch topology with device parameters.

The design process of output circuit network can be represented in Figure 8.

**Figure 9:**
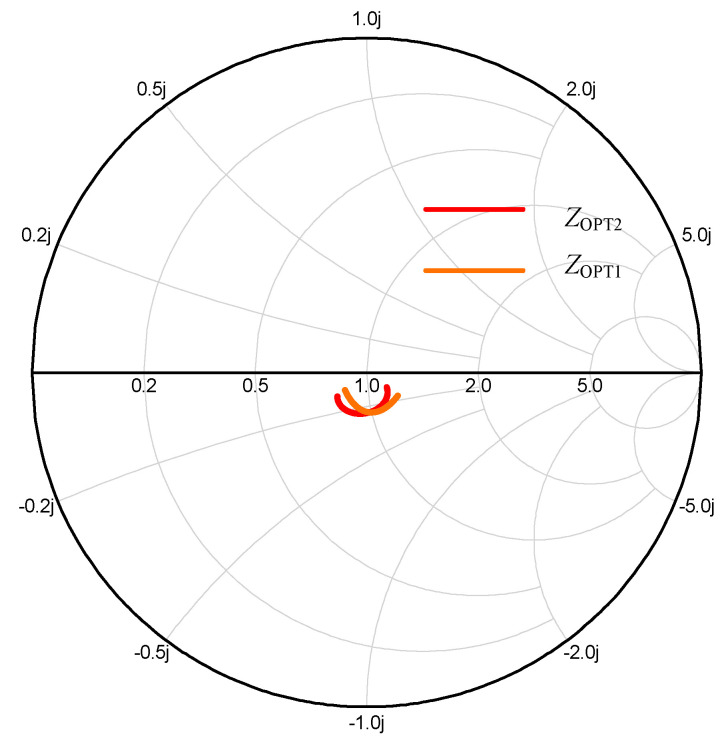
Simulated impedance *Z*_OPT1_ and *Z*_OPT2_.

**Figure 10:**
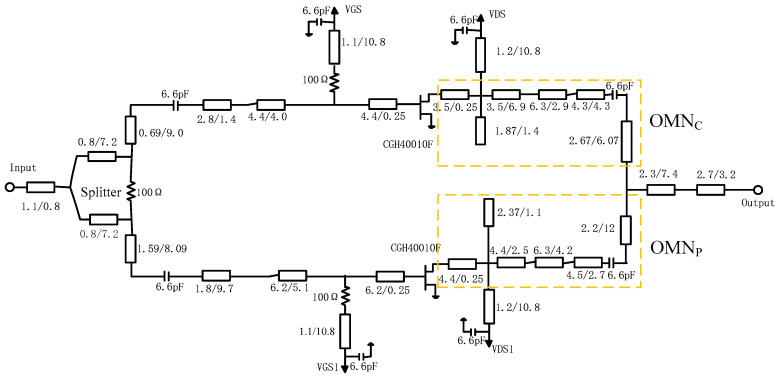
Completed circuit schematic and parameters value.

Firstly, the OBO should be determined; in this work, a 10 dB OBO was chosen. Then, load impedance *R_OPT_*, *γR_OPT_* of carrier and peak device can be calculated using Equation (15), respectively. The value of *γ* was 0.85. The load impedance at the combine node *Z*_L_ should be set, and was determined as 15·(1 + 0.9) Ω. Thirdly, load-pull simulation should be used to derive the *C*_DS_ and the package parameters of transistor. In this work, a general transistor CGH40010F was taken, and its parameters have been reported in previous papers. So, this step can be omitted. Micro-strip line TL1 and TL2 should be added to eliminate the package influence on accuracy of OMN, owing to the fact that ABCD transfer matrices of OMN are derived including *C*_DS_, and excluding package parameters. By selecting and optimizing the appropriate impedance and electrical length of the microstrip lines TL1 and TL2, the parasitic parameters can effectively be cancelled. Figure 9 shows the simulated value of impedances *Z*_OPT1_ and *Z*_OPT2_ that are labeled in Figure 7. It can be seen from Figure 9 that *Z*_OPT1_ and *Z*_OPT2_ have no large deviations. It confirms that the parasitic parameters are effectively eliminated by using microstrip lines TL1 and TL2. Then, the parameters value of (OMN)_C_ and (OMN)_P_ can be calculated based on Equations (17)–(24). The designed (OMN)_C_ and (OMN)_P_ based on the obtained values are shown in Figure 10.

### 3.2. Input Matching Network Design

Stepped impedance matching technique is used to synthesize suitable input matching networks and provide a targeted saturation gain of around 10 dB. A resistor *R* was added to the gate dc bias circuit to ensure the stability of both transistors. Before the input matching networks of PAs, a 3-dB Wilkinson divider was first employed to split the signal. Offset lines were also added in the input networks, to ensure that the signals of the carrier and peak branches were in phase at the combine node. Its circuit schematic is also shown in Figure 10.

### 3.3. Post Matching Network Design and DPA Overall Circuit Optimization

**Figure 11:**
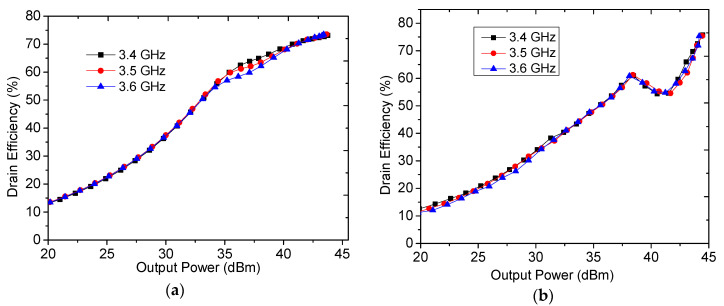
Simulated drain efficiency of DPA versus output power: (**a**) proposed DPA with 0.85 *R_OPT_*, and (**b**) conventional DPA with *R_OPT_*.

**Figure 12:**
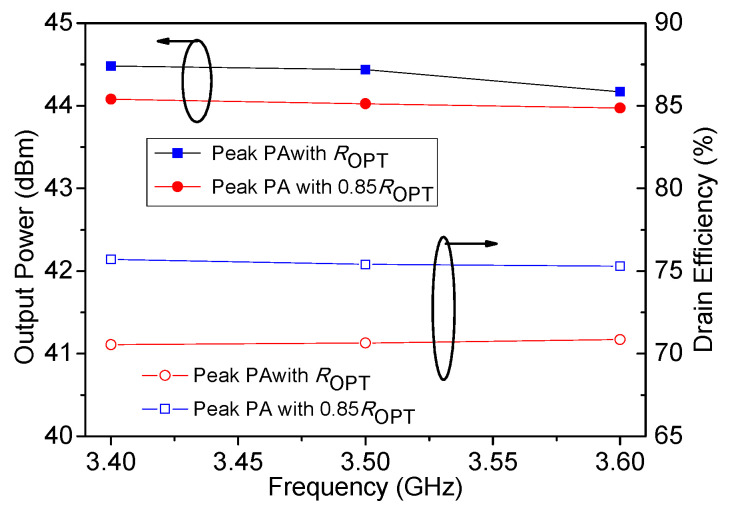
Simulated drain efficiency and output power of DPA in saturation.

As mentioned before, the load impedance *Z*_L_ was set to be 15·(1 + 0.9) Ω. Post-matching circuits should be designed to enable the load impedance to be matched to the 50 Ω standard. After all networks were designed, these circuits were combined into a completed DPA. The circuit of distributed parameters was as shown in Figure 10. In this paper, optimization was done in the Advanced Design System (ADS) software to improve performance. Simulated results are plotted in Figure 11 and Figure 12. Figure 11a,b display the drain efficiency versus output power for the proposed DPA and the conventional DPA at 3.4 GHz, 3.5 GHz, and 3.6 GHz, respectively.

Figure 11a,b show that the proposed DPA with load impedance 0.85 *R_OPT_* of peak PA has a larger OBO compared to that of the conventional DPAs with load impedance *R_OPT_* of the peak PA. This verifies the effectiveness of the proposed method. It also can be seen from Figure 11a that around 55% drain efficiency can be obtained at the 11 dB power back-off. Regarding saturated output power and drain efficiency, these are reduced by about 13% compared with that of conventional DPAs as shown in Figure 12. These simulated performances of the DPA validate the previously described theories. 0.85 *R_OPT_* of peak PA is selected, to sacrifice a certain amount of output power and efficiency in the saturation state in exchange for larger OBO.

**Figure 13:**
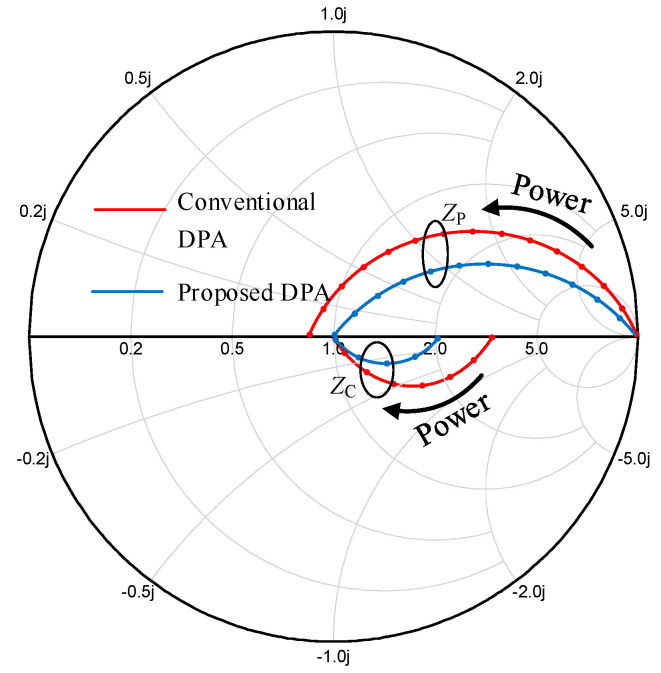
Simulated internal drain load impedance trajectories of the carrier and peak PA (normalized to *R_OPT_*).

Figure 13 shows the simulated impedance traces that are consistent with the theories. As shown in Figure 13, in saturation, the load impedance of the peak PA is about 0.85 *R_OPT_*, which is close to the theoretical value. The load modulation trajectories of the carrier PA also illustrate a larger OBO range compared to that of traditional DPAs. The load modulation trajectories of the proposed DPA are closer to the real axis, which means higher efficiency can be achieved, compared to that of the traditional DPA.

## 4. Experiment and Results Analysis

**Figure 14:**
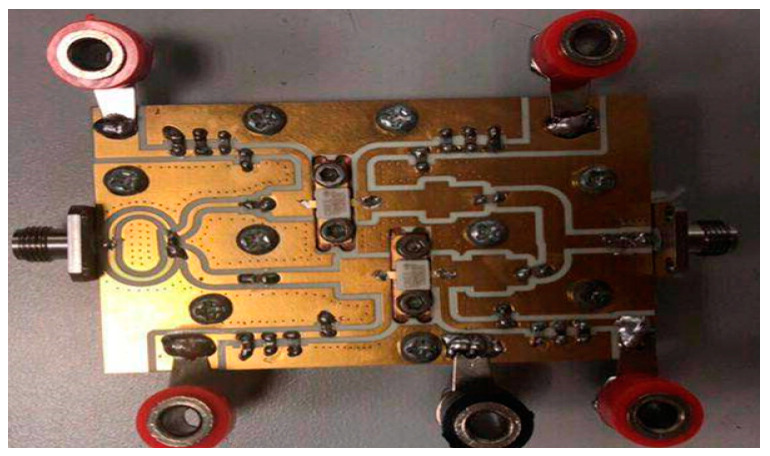
Photograph of the fabricated DPA.

**Figure 15:**
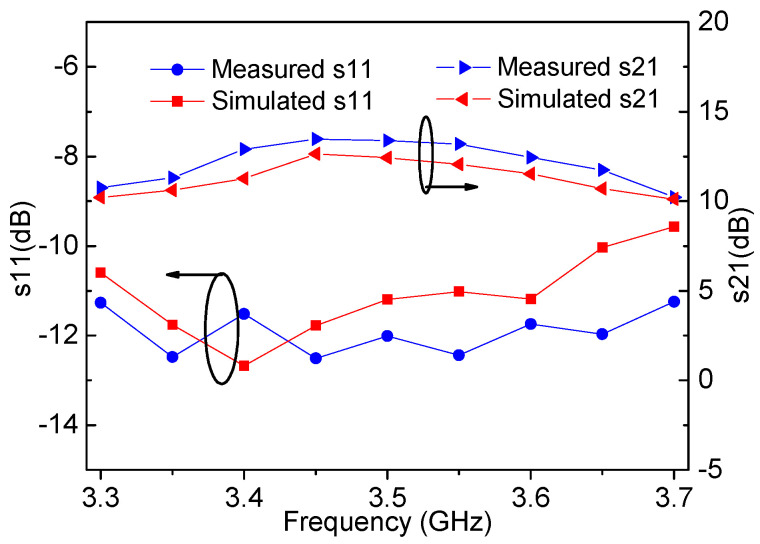
Simulated and measured small-signal frequency responses of the designed DPA.

In order to demonstrate the actual performance of the designed DPA circuit, a DPA was fabricated based on the circuit schematic designed in the previous section. Figure 14 is a photograph of the fabricated DPA. The small signal characteristics S-parameter are firstly tested. The simulated and measured *S-*parameters are plotted in Figure 15.

### 4.1. Continuous Wave Testing

**Figure 16:**
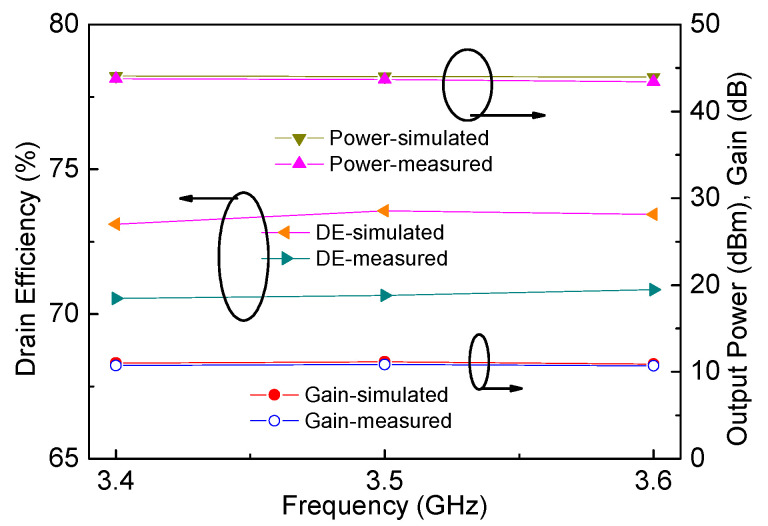
Simulated and measured output power, drain efficiency, and gain in saturation.

**Figure 17:**
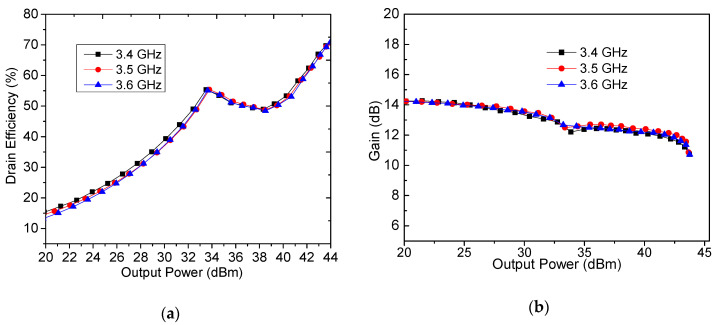
Measured drain efficiency, gain, and power added efficiency (PAE) versus output power: (**a**) drain efficiency, and (**b**) gain.

Performances of the designed DPA were tested using continuous wave signals. Measured output power, drain efficiency and gain are plotted in Figure 16 and Figure 17. Seen from Figure 16, it can be observed that the saturated output power is 43.4–43.7 dBm, and the saturated drain efficiency is 70.5–70.8% in the frequency range of 3.4–3.6 GHz, while gain is between 10.7 dB and 10 dB. Figure 17 shows that drain efficiency at the 6 dB power back-off is 62.4–64.3%. At the 10 dB power back-off, drain efficiency can be larger than 50% (50.3–52.6%).

In order to allow comparisons with previous reports on symmetrical extended efficiency range DPAs, Table 1 lists the performance reported in the relevant literature and the DPA designed in this paper. Except for reference [27,28], the OBO obtained by this work was larger than that of the listed papers. In fact, the drain efficiency at the OBO is a lot smaller than some listed papers, such as [17,19,21]. Compared with [27,28], the drain efficiency in this work is better than that of reference [27,28], with 10 dB OBO achieved. Moreover, it should be noted that the operating frequency is 3.5 GHz, which is higher than others reported in the literature. It is clear that the proposed DPA has higher operating frequency and OBO, making this DPA more suitable for 5G communication applications. The realized DPA has a high efficiency range of up to 10 dB, which is conducive to the wider development and application of DPAs in 5G communications.

### 4.2. 20 MHz 9.5 dB LTE Testing

**Figure 18:**
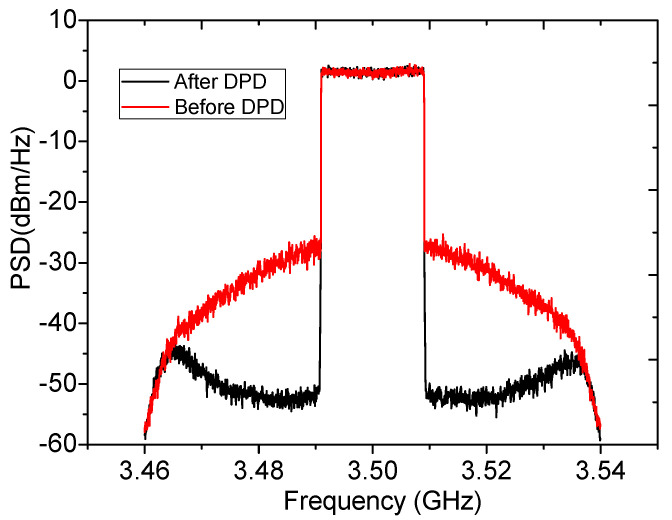
Measured adjacent channel ratio (ACLR) value.

In order to characterize the linearity of the implemented DPA, the adjacent channel ratio (ACLR) was tested by using an LTE signal with a bandwidth of 20 MHz and peak-to average ratio of 9.5 dB. The measured ACLR with an average output power of 34.0 dBm is plotted in Figure 18. It can be observed that the ACLR is better than −29.4 dBc at 3.5 GHz. After adopting digital pre-distortion technology (DPD), the ACLR value is better than −53.6 dBc.

## 5. Conclusions

This paper proposes a Doherty power amplifier with a large high-efficiency range. Theories of DPAs with *C*_DS_ included are derived, in which a new way was found for extending the efficiency range of DPAs, through selecting proper load impedance of the peak PA. An extended efficiency range DPA is successfully designed and fabricated by using two equal transistors based on the proposed theories. Measurement results show that the designed DPA can deliver over 43 dBm output power with drain efficiency of about 70% in saturation. Furthermore, at the 10 dB power back-off level, the drain efficiency is greater than 50%. More importantly, all these properties are obtained when the operating frequency is 3.5 GHz. These features indicate that the designed PA could be successfully applied in 5G communications in terms of operating frequency, high efficiency range, and linearity.

## Figures and Tables

**Table 1:** Performances Compared with Recent Symmetrical DPAs.

Ref.	Freq (GHz)	Pout@SAT (dBm)	DE@SAT (%)	OBO (dB)	DE@OBO (%)	PAE (%)	Gain (dB)	Tech	Bandwidth (MHz)
2014 [17]	2.0	42	67	8.7	57	N/A	10.4	GaN	100
2016 [18]	1.95	44	68	9	47	43	9.5	GaN	200
2017 [19]	2.1	42	72	9.5	58	N/A	10.2	GaN	250
2017 [20]	2.3	45	68	9	49	N/A	11	GaN	100
2019 [21]	2.2	43.6	71	9	54	50	9.7	GaN	200
2018 [22]	1.7	42	71	9	53	N/A	N/A	GaN	350
2019 [28]	2.2	44	69	10	45	N/A	10.5	GaN	200
2019 [29]	3.1	44	71	10	44	N/A	10	GaN	400
T. work	3.5	43.7	70.8	10	52.6	48.7	10.7	GaN	300
